# An Essay on Scrofula, Submitted to the Medical Society of Tennessee, at Its Annual Meeting in May, 1846*Note.—The Medical Society of Tennessee awarded to this Essay a premium of fifty dollars.

**Published:** 1846-11

**Authors:** W. L. Sutton

**Affiliations:** Georgetown, Kentucky


### WESTERN JOURNAL

OF

### MEDICINE AND SURGERY.

NOVEMBER, 1846.

---

ART. I.—*An Essay on Scrofula, submitted to the Medical Society of Tennessee at its annual meeting in May*, 1846.* By W. L. SUTTON, M.D., of Georgetown, Kentucky.

When we reflect on the immense amount of suffering occasioned by Scrofula in its endless variety of forms,—that from a sixth to a fourth of the human race is carried off by it, a sufficient reason why it should be regarded by the medical profession with intense interest, becomes at once evident. We accordingly find that some of the brightest geniuses in the profession have spent much time in investigating its causes, nature and treatment. After the labors of a Laennec, a Portal, a Louis, an Andral, a Lugol, a Carswell, a Stokes, a Graves, and various others of equal standing in Europe, to say nothing of their compeers in our own country, it would seem

*NOTE.—The Medical Society of Tennessee awarded to this Essay a premium of fifty dollars.

that little could be left to be learned; or if anything were left, that the hopes of success were faint indeed after they had failed. But the importance of the subject is so great, the benefit resulting from any improvement in correctness of views, either of the causes, pathology, or treatment, so widespread, that we are imperiously required to continue our research, notwithstanding all discouragements. Nothing was ever made by despair; on the other hand, circumstances which, upon full examination, have appeared utterly hopeless, have yet been retrieved by perseverance, aided by well-directed efforts. In this state of the case, not pretending to know more of the disease than my fellows—on the contrary, duly sensible of the difficulty of the undertaking, and of my own inability to do anything like justice to the subject, I yet propose to offer a few remarks upon this important disease. I do this because, although I am not able to offer anything new respecting the complaint, yet if I look at the subject in a light in any degree different from others, or even arrange my ideas in trains somewhat different, even that may have the effect of stimulating the enquiries of others, and good may result. I have reason to hope, then, from the difficulties admitted to belong to the subject, and the liberal feelings of the profession, that this humble effort of an obscure member of the profession may be received with indulgence.

It not unfrequently happens that words, even professional words and phrases, are taken in very different lights by different members of the profession. Perhaps this is as true of *Scrofula* as of any other word or phrase. It is important, then, that each man delivering his opinions upon this subject should take some pains to show in what sense he understands the term; and it is equally important that his readers should keep the writer's definition in view, else, whilst reading, he may apply the terms to states of the system very different from that which the writer intended.

I shall divide my subject into three heads, *Scrofulous Diathesis*, *Scrofulous Affections*, and *Scrofula.* I am fully sensible of the objections which lie against this division, as well as the difficulty of keeping the heads separate; yet am I equally sensible, that some such division is necessary, and that, in the main, this division is right.

As regards the scrofulous diathesis, I apprehend I shall not differ from those who recognise its existence as distinct from scrofula itself. It is usually marked by thinness and delicacy of skin, having a sufficiency of redness, perhaps, but yet a tinge of white not altogether agreeable to the practised eye; a thickening of the upper lip and of the columna nasi, also of the edges of the eye-lids; eyes very frequently blue, the conjunctivæ covering the scleroticæ, having a tinge of the same color; with usually a want of firmness of the muscular parts of the body, or rather, perhaps, a slight intumescence and want of firmness in the adipose substance. There is frequently an enlargement of the joints (particularly of the fingers, the ends of which are enlarged or clubbed), with a real or apparent diminution of the shafts of the long bones; a tumid abdomen; an inability to endure much bodily fatigue.

Such, in general, are the features of the scrofulous diathesis; but we find a strong tendency to scrofula in persons in some respects differently marked; for instance, we see those of dark skin, and black hair and eyes, frequently suffer with the disease.

Such persons, whilst this state of things continues, cannot with propriety be said to be diseased, much less to have scrofula. Under a favorable of train circumstances, they may even pass through life without becoming diseased. Yet such persons certainly are more liable to have the disease produced by a train of unfavorable influences than others. Therefore, we say that they have a scrofulous diathesis, tendency, or predisposition.

*Scrofulous Affections.*—On the score of diathesis, I do not presume that there will be any controversy; but I am not sure that I shall have the united suffrages of the profession upon what I shall say respecting scrofulous affections. By this phrase I mean certain disorders occurring in those of a scrofulous diathesis, which, although not in themselves indicative of scrofula, are more apt to arise in such habits, or are materially modified by this diathesis.

In this class I put certain eruptions of the scalp or skin in general, lice in the head or upon the body, intestinal worms, imperfections in various parts of the body, malformations, inflammations, &c. All of these affections may occur in persons who are not scrofulous; but some of them are much more apt to occur in persons of that tendency. We see a child with a dirty, scurvy head, and say the mother is a slut, and careless of her children. It may be true. It may however be true that the head of that child is affected with a scaly disease, which requires a great degree of care to keep it in tolerable order. Lice may be found in the head of any child; but it has been known from time immemorial, that some children are much more infested with them than others. Upon noticing such children, they will generally be found to have the characteristics of the scrofulous diathesis. The remark applies to those infested with intestinal worms. So true is this, that a thick upper lip, tumid belly, and pallid countenance, are, in popular estimation, constantly associated with the idea of worms. These, however, are amongst the most constant attendants upon this predisposition. We will find, too, by careful and extensive examination, that various imperfections of the body, as hare-lip, spina bifida, &c., although not confined perhaps to the scrofulous diathesis, are much more apt to occur in habits of that character than in those of the reverse. In the course of the last eight or nine years I have attended at the birth of two anencephalus infants, and in each case there was a scrofulous taint in the family. I do not now remember a single case in which I have operated for hare-lip, where there was clearly no such taint. On the contrary, in some cases, it was decidedly evident. In every case, too, of spina bifida, which has come within my knowledge, there has been good grounds for suspecting such a predisposition. The same was true of a case which I saw in which there was a deficiency in the walls of the abdomen, constituting what, for want of a better name, I called a *spina bifida of the navel.* This coincidence is the more remarkable, because in this community scrofula is by no means a frequent disease, compared with its prevalence in other countries.

Inflammation is not, I think, more apt to occur in scrofulous habits than in others; perhaps even less so. Still it may occur in such habits without, as I conceive, scrofula being present. I am fully aware that many, perhaps most physicians will be disposed to question this. I shall, therefore, take some pains to be understood upon this point.

Inflammation, when once set up, has a termination by resolution, effusion, suppuration or gangrene. If it end in resolution or gangrene, we are not apt to suspect scrofula; but suppuration or effusion may attend scrofula. The question then occurs, is there any difference in the course of inflammation occurring in scrofulous habits, and that formed by scrofula? I think there is. We have an abscess formed, upon the neck if you please; upon its being opened, it discharges what surgeons call a laudable pus, a yellowish white fluid of uniform consistence and color, so far as it is unmixed with blood, and of a consistence something thicker than cream. This case I should call one of *simple inflammation*, if it occurred in a habit untainted by scrofulous diathesis—*scrofulous inflammation*, if in one thus tainted. It may be asked, why in one case I call it simple, and, in the other, scrofulous inflammation? I answer, that although very similar, the cases are not identical. That which occurs in the scrofulous constitution will be found to be materially modified by its presence; there will be more tumefaction, less heat and pain, and the process of suppuration and cicatrization will be more tardy than in an inflammation producing the same quantity of pus, occurring in a healthy constitution. Abscesses thus originating, although longer in healing, are not apt to degenerate into ill-conditioned ulcers. A person of a strumous diathesis may have abscesses of this kind at divers times, yet not have scrofula developed in his system. But I was about to shew the distinction, in my opinion, between the product of *scrofulous inflammation*, and that of *tubercular inflammation*. Instead of the pus above described, tubercular inflammation produces a pus of unequal density, a part being made up of curdy, flaky, or granular portions, the remainder of a fluid more or less serous. One portion being decidedly more consistent than healthy pus, the other as decidedly less so. This difference, I hold, is caused by the presence of tuberculous matter along with the product of inflammation. It will be observed that I have used the terms *scrofulous* inflammation and *tuberculous* inflammation in contradistinction. I am not aware of any authority for so doing. So far indeed is this from being the case, that writers upon scrofula define by scrofulous inflammation just what I propose to define as tubercular; leaving what I call scrofulous inflammation undesignated, or rather confounding it with the other. Thus, 'in the commencement of scrofulous disorders, it deserves also to be remarked, that the inflammation which occurs has often so great a resemblance in its symptoms to simple inflammation, as to render it difficult, if not impossible, to distinguish between these two states.' [Thompson on Inflam. p. 106.] Again: 'The pus in these (scrofulous) abscesses is often serous and curdy, though in many instances I have found it quite healthy in its consistence and color.' [Ib. p. 128.] In the first quotation there is an implied acknowledgment of a difference between simple inflammation, and that occurring in 'scrofulous disorders,' with a directly acknowledged difficulty in marking that difference; in the other, there is, I think, a confounding of two states materially different. At least there is no attempt to show any cause for the different effects resulting from the same cause. I shall, therefore, give my reasons for it. I think it necessary that there should be a term distinguishing inflammation as occurring in scrofulous habits, from that occurring in those usually denominated healthy. It is true, we might use the phrase 'inflammation occurring in the scrofulous diathesis.' But this would be more circumlocution than we would like to repeat often. I have, therefore, proposed to appropriate to this state the term

'*scrofulous inflammation.*' But it is necessary to have a term designating that inflammation which occasionally accompanies scrofula, properly so called; I propose to apply to it the term '*tubercular.*' Not that tubercular is different from scrofulous, iflammation, but that both these states occur in scrofulous constitituons, one necessarily implying the existence of scrofula, the other only a predisposition to it. Just as we have words in English which have come to mean distinct things, though originally there was no difference—e. g. *transfer* and *translate.* So, too, we have a want of precision in some professional terms, which ought to be obviated. I will instance the phrase 'compound luxation,' which is used to signify two states very different—one a luxation of a joint, attended by a fracture of a bone connected with that joint, as of the ulna or fibula, in luxations of the wrist or ancle; the other a luxation attended with protrusion of a bone through the integuments. If this distinction should not appear satisfactory to my reader, I hope, nevertheless, he will keep it in mind, and when a more satisfactory nomenclature shall be invented, I shall always be ready to adopt it.

*Scrofula.*—By scrofula I mean the formation of tubercular matter on or in some part of the body. Whenever tubercular matter is produced upon any of the mucuous surfaces, or in any texture of the body, then is scrofula present. Before that takes place, there may be a strumous diathesis, or scrofulous affections; but scrofula, properly so called, is not developed. I know full well that some of the highest authorities in the profession are against me as to this division, at least a fair interpretation of portions of their writings would so indicate. Yet are there others of equal authority which countenance this as the true definition of scrofula. Further, some of those who appear against it, at other times appear to recognise it. Thus Lugol, who certainly appears to have paid as much attention to this subject as any other man, and who ought to have clear ideas upon it, says in a lecture delivered at the Hospital St. Louis, on *tubercular scrofula:* 'This is undoubtedly the most important form of scrofula; tubercles co-exist with all other forms of scrofula, they are the emblems of the disease—they are scrofula. We can only say that the existence of this vice, whatever it may be, is always congenital, and is always revealed by the development of tubercles; in fact, this production is scrofula itself, its anatomical and pathognomonical sign; this alone characterizes it and gives value to all the other symptoms.' *Lugol on Scrofula, preface, p.* 9. West. Jour. Med. and Surg. vol. 5, p. 285—from New York Medical Gazette. Again: 'Now *tubercles* and scrofulous *inflammation* occur very continually in the *same individuals.*' *Watson's Prac. p.* 112. 'A scrofulous abscess will form in the glands of the neck, and pus and tubercular matter will be discharged.' Ib. p. 116. Again: 'Next tuberculous or strumous disease is extremely common in the digestive organs.' Ib. p. 117. Again, Alison, as quoted in Cooper's Surgical Dictionary, says: 'In most cases in which scrofulous diseases are fatal, the diseased action is in internal parts, and the first symptoms are obscure and equivocal. The chief and certainly most characteristic appearances on dissection are tubercles in different stages of their progress.' Page 294. Lastly, MM. Rilliet and and Barthez, in a work upon the diseases of children, said to be of great merit, have labored to 'prove the *identity of scrofula and tuberculization.*' Med. Chir. Rev., Oct., 1845. By *tubercular matter* I mean 'a pale yellowish or yellowish gray, opaque, unorganised substance,' frequently of the appearance and consistence of half-dried mortar, at other times in small particles mixed or suspended in pus.

*Causes of Scrofula.*—Having, as I hope, given a clear exposition of my views on the division of the subject, I will now speak of the causes. First, I will take up hereditary descent as a cause of scrofula. That children are sometimes born with scrofula upon them, I am well satisfied; but most commonly they only receive from their parents a predisposition more or less strong. Whilst admitting the existence of a predisposition inherited from parents, I must beg leave to observe that I think greatly too much stress is laid upon it, as a cause of disease. Thus Lugol: 'So deeply does scrofula modify the whole organization, that persons afflicted with it may be considered as a variety of the human race.' Lect. Hosp. St. Louis—West. Jour. Med. Surg., vol. 5, p. 285. Those, too, who have read his late work on scrofula, will be aware that he inculcates forcibly the opinion that the disease is transmitted by hereditary descent; that a man free from the disease cannot acquire it himself, but may acquire a predisposition and transmit the disease to his offspring, who, of necessity, transmit it to theirs, until in three or four generations the family becomes extinct. In a population where scrofula is very common, it is perhaps more difficult to arrive at correct ideas as to the precise influence of particular causes, than where it is rare. It so happens that I am acquainted with the history of one family for five generations, which, it appears to me, throws some light upon the influence of some of the causes of scrofula. D. C., having enjoyed good health, lived to the age of 109 years. His wife, also of good general health, died at an advanced age. Their son, D. C. 2d, died middle-aged of bilious colic, leaving a number of sons. His wife, in good health, lived to an advanced age, having raised all her children, one of whom was an infant at her husband's death. Thus far, there is no appearance of any form of scrofula, nor of any of the causes which are enumerated as giving rise to a predisposition. Of their children, 1st, J. C. married and lived to middle age, a healthy, stout, industrious, and ambitious man. After a hard day's work on a hot day, (a heavy rain and considerable diminution of temperature taking place in the evening,) he took cold, and afterwards died of consumption. His wife, E. C., is now alive, aged about 55, in good health. He had two children who died of consumption. One born before the day's work which was supposed to have caused his death, the other after. He has a son now 35 years old, stout and in good health. 2d. C., now 75 or 80 years old, active and in good health. His family healthy, no chronic disease among them. Several other brothers lived to the ordinary rates of life, without any manifestation of scrofula. D. C. 3d, is now about 65 years old, married and had eight children. Of these, three are now alive, of the ages of 36, 34, and 18, in good health. One died when young of a violent injury received; one at the age of 35, of ulceration of the bowels, occurring in typhoid fever; one at the age of 34, of consumption; one at the age of 20, of consumption; and one at the age of 17, of consumption. Of the eight children, three only have married, two of whom are now dead; one of these lived in wedlock eight years, leaving two children, now 5 and 7 years old, in good health; the other lived in wedlock four years, leaving one son, now 4 years old in good health. The third married into a family tainted with scrofula, has five children apparently healthy. The mother of these eight children died suddenly at the age of 55, having, as was supposed, labored under a hepatic derangement for a long time, say from the age of 30 up. For a number of years prior to death, she enjoyed an improved state of health. She had been affected, however, for a number of years with what appeared, from its history and condition when I first saw it, to be an enormous bronchocele. Her sister, who also was wife to J. C. above named, is now alive, about 55, and also affected with a similar tumor, but not so large. Several of the children of D. C. also have had slight enlargement of the thyroid glands. They live, however, in a locality where that affection is more general than in any other region within my personal knowledge.

The father of these two Mrs. C.'s, Mr. H., lived to the age of 85 years, having generally enjoyed good health. For about fifteen years before his death, he had been sensible of a tumor of the size of a filbert in the middle of the right side of the neck, supposed to be an indurated cervical gland. About six months before his death, this tumor commenced enlarging very rapidly, and at his death, which appeared to be caused by it, it was nearly as large as his head, having pushed the trachea very much to the left, the thyroid cartilage being in a line with the left ear. Before death he had expressed a wish that it might be removed after his decease. I complied with his request, and found the tumor to embrace full half the calibre of the thrachea, notwithstanding its displacement, and to enclose completely the right carotid and subclavian arteries. This tumor was mostly of a cartilaginous consistence; in part approaching suppuration; but in no portion of it was there anything of a tubercular character. His wife, Mrs. H., is still alive, about 80 years old, and as healthy as persons of that age can reasonably expect to be. In addition to the two Mrs. C.'s, they had one son, now about fifty years old, who has suffered extremely with rheumatism, having been confined to his bed for eight or ten years at one time with it, and again for the last five years. Nothing of a tubercular character having affected him at any time. He has raised a large family of children, who, thus far at least, have enjoyed good health. His wife died five years ago, having previously requested that her body might be inspected after death, in consequence of several of her family having died similarly affected. Accordingly the late Prof. Richardson and myself attended to that duty. We found her liver enormously enlarged and diseased, a large amount of fluid in the abdomen and also in the thorax. The stomach externally appeared healthy, but on the internal surface, near the pylorus, were two tumors, one as large as half a walnut, the other as large as half a nutmeg. Lungs healthy; no appearance of tubercular matter anywhere. I have introduced the history of this family because it comes to me in a perfectly authentic form, and extends farther back than we can often trace families with certainty; and because it appears to me to bear on several points respecting hereditary diseases. My opinion is that scrofula is very generally transmitted from parents to offspring; but I do not believe that the child of a scrofulous father or mother will *necessarily* be scrofulous; nor that a child, whose father and mother are free from the taint, will *necessarily* escape scrofula. Children generally have a greater or less resemblance to the father or mother, and the child that resembles the father will be more liable to be affected with that disease to which the father is more liable. The same principle is true of that child which resembles the mother. If, then, both father and mother are liable to the same disease, the children will be very apt to suffer from it. But it occasionally happens that a child does not favor either of the parents. Such child will be less likely to suffer from any disease to which the parents may be predisposed. Or if both parents are healthy, he may by his conformation be born with a predisposition to some disease, till then unknown in the family. This will readily be admitted by certain physiologists as to the reputed parents; but they will say that it is not true of the real parents. Thus M. Lugol in such cases calls in the aid of adultery to maintain the truth of his theory. Some men have very little confidence in the chastity of females; and Lugol, residing where adultery is confessedly common, may be excused for his scepticism. In this country, however, cases of *crim. con.* are very rare, and I am satisfied that adultery is not the besetting sin of our ladies, yet the same facts are observed here as at Paris. Neither do I see why we should reject a law respecting mankind which is known to be true as to brutes. We will take for example the hog. A male and female will produce a litter of some eight to twelve. It may be that the whole litter will resemble the sire in form; or it may be they will all resemble the mother; or a part may favor one, and a part the other; or, again, they may partake of the form of both. It is quite likely that some one or more of the pigs may be found in each category. Again, now and then a pig is found having no appreciable resemblance to either. By taking advantage of such cases, and using some care, a man can in a short time completely change his breed of hogs, without any extraneous cross. The same is true of other animals.

But certain conformations and appearances of the body are found to be attended by predisposition to certain diseases; and as such conformation is similar to that of the parent, and as the parent is liable to the same disease, the predisposition or the disease is said to be inherited from the parent. This is as true of some other diseases which are not considered hereditary, as it is of scrofula. The family to which Mrs. H. above mentioned belonged, was predisposed to hepatic disease. I have seen a number of families in which, I was clearly satisfied, the same affection was transmitted from parent to child. Again, a certain conformation is known to predispose to apoplexy. A son frequently gets this conformation from his father, and with it a liability to the disease. But nobody considers hepatic disease or apoplexy as hereditary. This principle is very clearly elucidated in the history of various females brought forward by Lugol in his work on scrofula, in which various members of a family are found affected, with not only the same disease, but in the same form, and affecting the same parts.

It will be seen, that whilst I believe that very generally scrofula is transmitted from parents to offspring, I do not believe it to the same extent as some others. I do not believe that a man is necessarily scrofulous because his father or mother and divers brothers and sisters are so; nor the converse. I have endeavored also to give my rationale of the matter. Whether there is any difference between my views on this point and those of others, I am not prepared to say. In the family, the history of which is detailed above, there is no appearance of scrofulous affections for three generations on the father's side, and none for two on that of the mother; unless the bronchocele should be so considered. This affection was at one time generally considered scrofulous; but I believe the opinion is losing ground. There is a region of country in this vicinity in which bronchocele is quite common; and it certainly appears in persons who have no appearance of scrofulous taint. It is also true that there lived a family in that region which appeared predisposed to scrofula, none of whom, so far as my knowledge extends, was troubled with this tumor. It may be noted, however, that the two daughters of Mr. H. were affected with it, and each had children who died of consumption. But it may be remarked further, that the tumor of which the father died, at the time I saw it, only a few days before his death, resembled very strikingly those on the necks of his daughters, only being greatly larger. It is possible, too, that in the daughters these tumors may have commenced in some situation other than the thyroid gland. His tumor differed also from theirs, in remaining long stationary, and then growing very rapidly; and also in showing some disposition to ulcerate. It is true, however, that the children of Mrs. S. C. who had swellings on their necks, had them in the situation of the thyroid gland. None of those thus affected have shown any disposition to consumption or any other form of scrofula. Now did those children of D. C. and S. C., who died of consumption, get their predisposition from their parents? I think not; and this opinion is, I think, justified from the facts detailed. With the first who died, I had little acquaintance, and will therefore say nothing. The second was decidedly leuco-phlegmatic in temperament, a good deal disposed to bloat, digestion bad; apt to be troubled with flatulence; menstruation came on tardily and imperfectly. She appeared to be improving under the use of iron and iodine, when she was seized with a violent attack of typhoid fever, from which she imperfectly recoved only to have pulmonary consumption fully developed; from which she died in six months. The third did not appear to be so much predisposed, but was of slender delicate frame, and appeared to have contracted her disease from exposure to cold.

But it is said that a man who has never been affected with scrofula, or even predisposed to it, may have a predisposition generated by various diseases, as syphilis, &c., and that children born of such parents, after such event, will have scrofula developed in them; whilst those born previously will not. Lugol, in his work on scrofula, gives several instances in which the disease was transmitted in this way. I must confess, however, that I do not appreciate the force of his reasoning. In the first place, to say that a child inherits from his father that which his father never had, appears to me very much like a solecism. Again, if the transmission is of a specfic character, it seems to be wrong. We know that a man may have syphilis and be, to all appearances, cured, yet transmit that disease to his offspring, but not scrofula. Lugol's opinion appears to be different, if I understand him correctly. And lest I should misrepresent him, I will quote his words: 'But some years afterwards, his habits became dissipated; at that time, whilst exhausted and syphilitic, having also infected his wife, he had a third, who was born scrofulous.' Lug. on Scrof., p. 100. 'Syphilitic cachexia and after it scrofulous cachexia, have progressed much more rapidly in Spain, because the venereal disease has been generally neglected there, or is treated by remedies too inert to produce a radical cure.' Ib. 230. The same sentence is repeated on the 232d page. How these passages, as well as the general scope of his observations in his article on the syphilitic origin of scrofula, tally with the following sentence at page 167, each reader will judge for himself; for myself I must acknowledge they do not appear to me to agree very well: 'Scrofula also has relations with syphilitic diseases, and very frequently with erysipelas; but all the relations it may have with these diseases are only complications; they may favor its development, but it is certain they can never produce it.'

I can very well imagine that the injury inflicted on the constitution by protracted syphilis, and by the treatment necessary to cure it, may leave the body in a condition favorable to the evolution of scrofula; but I do not see how it would cause it of necessity; and I must repeat, if I have received his idea correctly, it is not in accordance with the experience of the profession in this country. If, however, it is meant that the constitution may be so much injured as to bring on premature decay, I agree to the proposition. Children born after this, may be of an imperfect organization—*may* be predisposed to scrofula, or *may have* it developed; but I do not believe they will *necessarily have* scrofula. I shall hereafter have occasion to express my opinion that the improper employment of mercurials is a very powerful cause of the development of scrofula. And it is not at all unlikely that much of the blame thrown on syphilis in this matter, is in truth due to the remedy used to eradicate it.

*The excessive use of mercury* is, I think, a more frequent cause of scrofula than a syphilitic taint. It is well known that an inordinate use of that mineral will produce a leucophlegmatic appearance of the face, attended with tumefaction of the lips and face generally. A superabundance of white tissues is developed, and a deterioration of the red globules takes place in the blood. Frequently, too, there is some tumefaction of the abdomen. In short, a state is produced very analogous to a strongly marked scrofulous diathesis. A person whose general health is good, may have this state produced, and it may pass away without the production of the disease; but if the powers of life are languid, he will run considerable risk, if this state continues any time, of having his health permanently injured, and scrofula may be produced. During the late mania in the use of calomel many persons had their health ruined; and not a few had scrofula developed, in whom no other assignable cause appeared.

Somewhat similar to the effects of undue mercurialization, are those of excessive bleeding, whether practised for the cure of disease, or as occurring in the form of hemmorrhage The consequence of great loss of blood is necessarily a want of a due proportion of red globules. The same anemic appearance takes place as is observed after excessive salivation. Indeed there is so great a similarity between the two states, that they may be considered identical.

There are numerous other causes which produce imperfect sanguification, and therefore are more or less active in predisposing to scrofula; but it is scarcely necessary to enumerate them, as I hope the principle which I wish to inculcate is sufficiently evident.

No *age* can be said to be exempt. The fœtus in utero is found affected, and the man of old age is occasionally afflicted. The disease is, however, more commonly met with from the age of two or three years to that of thirty or thirty-five. Although any form of scrofula may appear at any age, yet some forms are met with more frequently at some ages than at others. In early life, say under ten years, the skin, the nose and lips, the brain, and the lymphatic and mesenteric glands are most apt to be affected. From puberty to twenty-five the lungs are especially obnoxious in white persons; but I think that negroes are as apt to be affected in their abdominal viscera at this age as in the thoracic. I do not know how the experience of others agrees with mine, but I have found the osseous system more frequently affected in those from ten to fifteen years of age.

Both *sexes* are liable; and perhaps each is *per se* equally apt to have the disease developed; but our domestic habits, in my opinion, have an influence injurious to the female. Her sedentary habits, the want of a due exposure to air and sunshine, are calculated to exert an unfriendly influence upon her health in general, to prevent due vigor and activity in the chylopoietic viscera, and to render her unable to withstand sudden changes in circumstances calculated to affect the health.

Climate was at one time considered as particularly efficacious in developing scrofula; but it is found prevailing in all climates; so that we are compelled to exonerate particular ones from the opprobium of producing it. It is found, however, that a sudden transition from one climate to another materially different, is very favorable to the development of the disease. Neither does it matter, except in degree, whether this change is from a cold to a hot climate, or the reverse. The opinion that the removal of scrofulous, and especially phthisical patients to a southern climate is very favorable to convalescence, has been so common, that the idea advanced here will be by many considered as absurd. Many things, however, equally absurd in appearance, have been by strict investigation proved to be true. The best test upon this subject will be one in which satisfactory information as to the health of large bodies of men, in other respects similarly situated, can be had. Such test the British troops, situated in the United Kingdom and those situated in the West Indies, furnish. I quote from a statistical report made by the medical department of the British army to Parliament. Med. Chir. Rev., vol. 30, p. 59: 'As an instance how much more prevalent consumption is in that country than in Britain, we may state that of an aggregate strength of 51,567 serving in Jamaica, there have been 661 treated for that complaint, being at the rate of 13 per thousand annually; while out of an aggregate strength of 44,611 dragoon guards and dragoons serving in the United Kingdom, there have been treated only 286, or between 5 and 6 per thousand annually. * * * The baneful influence of the climate of the West Indies in accelerating the progress of consumption, has often been remarked by the medical authorities; but it does not seem to have occured to them, nor indeed had they the means of ascertaining, that at least twice as many cases of it originate as at home, though those catarrhal affections to which they are generally attributed are there comparatively so rare.' Again, p. 52: 'In respect to pulmonary affections, though the admissions under this head are less than at home in the proportion of 115 to 148, yet the mortality is greater, 10½ per thousand of the whole strength having perished by lung affections. The average per thousand in the United Kingdom was 8½.' 'In the West Indies, 1,023 cases of consumption occurred in an aggregate strength of 86,661; being 12 per thousand annually. In the United Kingdom the proportion was 5½ per thousand.' 'This,' says the reviewer, 'we should think may open the eyes of some physicians who think a hot climate a panacea for diseases of the lungs.' Page 59. Why should there be a greater mortality from consumption in the same kind of persons in the West Indies, than in England? I think mainly because of the highly injurious influence exerted upon the constitution by the great change in climate. There will be but little difficulty in believing that a change from a warm to a temperate or cold climate exerts a prejudicial influence on the health. In confirmation of it, I shall refer to two cases only, the great prevalence of scrofulous diseases in our negro race, and the equally frequent occurrence of them among the animals in the garden des Plants in Paris. I am not sure that I ever saw but one native of Africa; and he died of tubercular consumption. It is known, too, that the African race in this country suffer much more severely from scrofulous diseases than the whites.

A damp atmosphere has been thought favorable to the production of scrofula, but numerous observations have demonstrated that moisture alone is not sufficient to generate it. I can readily believe, however, that a cold moist atmosphere will be more favorable to its development than one of an opposite character.

Of the seasons, the latter part of winter, spring, and the forepart of summer, are found to exhibit the disease most frequently. Indeed it is very common for scrofulous affections to appear during this period, gradually disappearing during the summer and fall, and re-appearing the next spring; thus conforming to the seasons, for years. From this we infer, not that spring in itself is more favorable to the evolution of scrofulous diseases, but that cold is a powerful agent in its development. It may be asked, if cold is the active agent in this matter, how it happens that the increase in the disease does not take place during the cold of winter? My answer is, that many effects do not appear until long after their causes have passed away. The tides do not keep pace with the sun and moon, but follow them. The weather does not begin to grow warm at the winter solstice, but some six or eight weeks after. You may travel a horse two or three hundred miles, and at the end of your journey you may not be aware that he has lost flesh; yet will he fall off afterwards, though you use him not and feed him well. So the effects of the winter are generally produced, and do not appear in full force until after the cold weather has passed away.

This explanation appears to me more satisfactory than that offered by Lugol. 'It is only, says he, 'when there is a morbid predisposition that the animal economy is unfit to receive the salutary influence of the return of light and heat, and that this influence, instead of being profitable, lights up the germ of hereditary diseases, or favors their progress when they have already attacked.' Op. cit. 241. Again: 'So, too, with all scrofulous diseases in general, they seem to redouble their activity when nature awakes after the sleep of winter.' Page 240. Again: 'This recrudescence, considered generally, presents a movement of increase and decrease; it commences in the first days of January, it has its apogee in the month of March, and it decreases till the month of June inclusively. In nine-tenths of the scrofulous patients the disease increases during this semestrial period, which embraces the whole period of the year that the sun remains longest in our horizon.' Page 240. It appears to me, if the return of light and heat produced an invigorating effect upon the disease, rather than upon the constitution of the patient, that that increase in the strength of the disease should continue until fall or winter unabated—in fact, augmented. It seems to me strange that M. Lugol should say that this semestrial period from January to June embraces the whole period of the year that the sun remains longest in our horizon; when, in fact, the sun remains just as long in our horizon during the other 'semestrial period.' From March to September is 'the period that the sun remains longest in our horizon.' But the influence of the cold weather remains for a given period; the returning light and heat begin to exert their invigorating influence upon the constitution; the disease gradually feels this influence and begins to give way; and a gradual improvement of health necessarily takes place. By the bye, I hold this to be no mean argument against the idea that scrofula is an inflammatory disease.

But *imperfect nutrition* is perhaps the most potent of all causes in the development of scrofula. The heat and light of summer follow the cold of winter, and to a great degree repair the injury sustained during the latter named period. When the body has sustained a temporary detriment, as by profuse hemorrhage or salivation, there is a *vis medicatrix naturæ*, a restoring power in the system, which most frequently is sufficient, after a shorter or longer time, to bring it back to healthy action; but imperfect nutrition is an ever-acting, ever-powerful agent of evil, the existence of which, and the extent of which, it is frequently extremely difficult to appreciate. This cause may be divided into two modes of action, one caused by improper food, the other by a deficiency in the powers of digestion, by which even healthy food is not assimilated. There are few subjects in the treatment of disease upon which it is more important to arrive at the truth, or more difficult, than what is the quality and quantity of our patients' diet. Some persons are not satisfied if an invalid does not eat at least as much as a laboring man should; others again think an invalid should be starved to any degree short of death. Hence in one instance we will be told that the patient 'eats nothing at all,' and in another that 'he eats a plenty for a person in his situation.' Whereas when, by cross questioning, we approximate the truth, we may form a very different opinion. As regards climate, so of diet; a sudden change from a full to a spare diet, or even the reverse, is unfriendly to health, and especially so in those whose tendencies are towards scrofula. This may be accounted for, in the one instance, by the want of a sufficiently healthy nourishment, by which the body is imperfectly supplied with materials of renovation; in the other, by injury done to the powers of digestion, by taxing them beyond their strength, by which the same result takes place. It matters little how good the fuel is, if placed under circumstances so that it cannot burn, no heat is evolved.

Such are the causes which I esteem most prominent in the development of scrofula. Of some others adduced by Lugol, as excessive venereal indulgences, the husband being too old, or disproportioned in age to his wife, or not having the relative strength of his sex, &c., I do not see the force. The bringing forward of such causes appears to be a very strong evidence of the whims into which a man may be betrayed, whose attention is devoted almost exclusively to one subject. In reading that part of his book, I was forcibly reminded of a certain quack who declared his patient's disease to have been brought on by the use of mercury. But, doctor, said the patient, 'I have taken no mercury.' 'Did you not take a dose of calomel?' 'No.' 'Ah! your doctor gave it to you in disguise, for sure as death you've got it in you.' 'But I have taken no medicine at all.' 'I do not mean at this time,' replied the quack, 'but it may have been years ago.' 'I tell you,' answered the patient, 'I never took a dose of medicine in my life.' 'Did you ever have the itch?' 'No.' 'Nor ever rub mercurial ointment on you for anything?' 'Never.' After a pause: 'Have you eaten any beef in the last year?' 'Oh yes.' 'That is it; I knew I should get the clue after a while. That beef when a calf was lousy and mercurial ointment was rubbed on it, and you got the mercury by eating the beef.' Let me not be charged with a want of respect for Lugol. No man, who has labored as long and as diligently for the promotion of science as he has, can be held in indifference by me. I only wish to avoid being swayed by his influence beyond due bounds. In a population where scrofula is present on a large scale, a man may take any position in regard to it, and find circumstances to bear him out. One great difficulty in our profession consists in separating that which is true from that which is plausible.

I have introduced the history of a family for five generations, because, as then stated, I received the account in a way which satisfied me as to its correctness; and because it appeared to me to bear somewhat upon the causes of scrofula. The gentleman who gave me the information, and who is now about 65 years old, and one of the parties, removed to this country from New Jersey when a young man. He married here a wife who had also come from the same region as himself. No scrofulous taint was known in the family of either, and none occurred until the fourth generation on his side, and the third on the side of his wife, (as appears from the account of his family and my own personal knowledge of hers,) unless the bronchocele on the neck of his wife, and also on that of her sister, be considered such? Some will consider them as scrofulous, and the facts in the case of S. C.'s children may be adduced in support of it, viz: that some of her children had small enlargements of the thyroid glands, and some of them died of phthisis. Again, those who died phthisical, at least one of them, favored the mother's family. But there is another view to be taken of this matter. It is very common, as is abundantly shown by the observations of Lugol, that scrofula is generally observed to affect the different members of the same family in the same way. Now although those who died phthisical favored the mother's family, those who had goitre did not. I have said that I did not believe that this family was affected by inheritance. Others may perhaps think differently, and adduce the devolopment of the tumor on the neck of Mr. H. at his advanced age, as an evidence. Such may be the truth.

Here I will mention the extreme caution with which it is necessary to investigate the history of families; and as an instance is better than a description, I will give one. About a year ago, I was called to see a little boy who labored under some of the symptoms of coxalgia. As I was not acquainted with the family, I thought it my duty to go into an examination of its history. The mother was considerably nettled that I should suppose that there was any taint in her family. Yet upon investigation I found that several of the family had died of consumption; and she herself died of the same disease in less than a year. A friend of mine also highly affronted a whole family by saying that a case to which he was called was one of cancer. Self-love and family pride will hide many things from our eyes.

But to return to our history. This family was in easy circumstances, lived well, and had the usual comforts of the country. It is true that when they first removed to this country it was quite a new one, and much labor was necessary to open farms, &c. The different members of the family were of course exposed to all the circumstances attending the settlement of a new country, but to no inordinate degree. The change of climate had not been great, indeed within a range which would have been considered as exerting a prophylactic influence, if any. They were all of temperate habits; were guilty of no excesses of any kind. There was a suitable correspondence of age between man and wife. In short, there was no evident cause for the development of the disease. The good health of the family was like the 'common law' 'of which the memory of man goeth not back to the contrary.' Yet we have phthisis developed in the family. Why? Some will say because of the taint of the mother. But with the exception of these tumors about the neck, that branch enjoyed as good health as the other. I would say that disease appeared in the fourth generation, of which we have an account, because in that generation children were born having the marks of the strumous diathesis upon them, which were quickened into existence by exciting causes. This brings me to enquire, can a healthy man beget a scrofulous child? In a practical point of view there is another question nearly allied to this, inasmuch as it is frequently very difficult to determine under which to place a given case; but in their nature they are entirely different, viz: can a child born free from scrofula become so? We sometimes see children which can be pronounced scrofulous at birth, but very frequently there are no marks by which such judgment could be given. These last afterwards have the disease developed, and it may be a question, the parents being free from disease, whether they were born scrofulous, or became so after birth. I entertain the opinion that a child of healthy parents may be born predisposed to scrofula. I am fully sensible of the difficulties which lie in the way of coming to a correct conclusion upon this point. But reasoning by analogy from what is known to be true of animals, as adverted to in the forepart of this essay, and from facts so far as human observations can be depended on, I think the testimony is ample. Whilst Lugol, by the general drift of his work on scrofula, negatives the idea, he yet furnishes facts which go to prove it. Thus 'A. Bodin, in his statistics of the Department du Nord, says that the inhabitants of Lille have scrofulous offspring when they intermarry, which is not the case when they marry strangers.' Page 235. Again, speaking of a population intermarrying, he says: 'Hence the race was debilitated, and after a certain number of successive generations the scrofulous state ensued.' Ib. 234. Dr. Stokes says, in a lecture on scrofula: 'It sometimes, on the other hand, happens that healthy parents may have children of a strumous habit. This, however, is the rarest case.' Amer. Jour. Med. Science, vol. 19, p. 476.

The other question, viz: can a person, born healthy, acquire scrofula? is to be answered in the affirmative also. Lugol recognises the truth of this answer when he says: 'A scrofulous nurse never renders her nurseling absolutely scrofulous, but gives it only the predisposition to this disease, which is developd at a late period.' Op. cit. p. 101. Stokes, loc. cit., speaking of animals, says: 'If we look at those animals in which tubercles are found, we see that they are often those which have been brought from a hot to a cold climate, and kept in a state of confinement for a long time.' Lugol, too, speaking of animals, says: 'Thus, too, most of the foreign animals in our menageries die in a very short time from pulmonary tubercles.' Page 239. Now it would be truly strange if those engaged in catching wild beasts should get hold of those affected with tubercles alone, and let all the healthy beasts of the forest escape. I will make but one more quotation in support of this position. 'Butler was of a family in no manner a prey to scrofula; in spite of the ills of venereal debauch, his constitution rallied and overcame until angina from aneurism hindered him in the duties of his office. Until then, almost pampered with the goods and comforts of this life, he at length could no longer earn his daily bread; poverty, want and distress came upon him as the storm upon the goodly bark. He was shattered in health, and the ill habit presided over the economy, and acute tubercle settled in the lung.' Dublin Journal Med. Sci., vol. 20, p. 19.

Another question of vast importance to those affected with scrofula is, can a scrofulous parent beget a healthy child? Can the child of scrofulous parents be so managed as to afford a reasonable hope that it shall enjoy a fair amount of health? I think that prudence would point out to those who are decidedly of a scrofulous habit, the propriety of abstaining from marriage. The strong probability is, that notwithstanding their utmost care, their children will be born to disease and misery, and probably will, most of them at any rate, die young. Yet the children of such parents, at least where one is not affected by the disease, are not to be considered as beyond hope. Some of the children may reasonably be expected to conform in their organization to the healthy parent. By rigid adherence to rational training, too, much may be done to invigorate the constitution and ward off the approach of the disease. By putting the child to a healthy nurse, if the mother is tainted, by a free exposure to a pleasant dry atmosphere, by constant invigorating exercise, and by great attention to the chylopoietic viscera, much may be accomplished. Notwithstanding the gloomy prognostics which Lugol applies to all persons affected with scrofula, he yet admits the possibility of an improvement in the health of such families, when sufficient pains are taken to secure that end. 'In the most fortunate cases,' says he, 'those where we can believe that the diseases anterior to puberty are completely arrested, scrofula may be transmitted hereditarily, and it is not till after several generations and happy alliances that the scrofulous habit ceases to be perpetuated in families.' Op. cit. p. 86. 'So, too, parents born in communities where scrofula exists endemically, if they are affected with this disease, do not protect their children from it by emigrating to more exposed and healthy situations; on the contrary, they carry the disease with them. * * * This emigration does not bear its fruit till after several generations, and only in cases where the children have good nurses, and where marriages are made discreetly.' Page 231. Stokes advances the same opinion: 'But on the other hand we sometimes find scrofulous parents beget healthy children. This appears to be an anomaly, but it may be explained by the circumstances of the child having a good healthy nurse, living in a pure air and having comfortable warm clothing, all circumstances calculated to develope the red tissues and therefore strengthen the system. Thus a scrofulous taint may be completely worn out in a few generations.' Loc. cit.

If the preceding views be correct, the prospects respecting scrofula will be rendered in some respects darker, and in some lighter, than we have been accustomed to consider them. Whilst a source of danger not hitherto recognised is shewn to exist, a source of hope is equally established; no material change, upon the whole, being effected, except such as arises from a more definite and correct knowledge of the grounds of those dangers and hopes, and consequently a more reasonable reliance upon the means used to ward off one, and to secure the other.

*Pathology.*—It may appear somewhat absurd to speak of the pathology of a diathesis, a predisposition; but as this state cannot be said to be one of pure health or of disease, I do not know any term better suited to express the idea.

Upon looking at a person having a strongly marked strumous diathesis, we are sensible of an aspect of countenance materially different from what is common. There is in certain parts an undue tumefaction; the color of the face is either not sufficiently red, or there is not a suitable distribution of that color. The cheek is perhaps sufficiently colored, perhaps rather too much so over a certain portion, but it is too circumscribed, or it is more or less marbled, especially after exposure to the cold; the remainder of the face being disagreeably pale. The eye may be animated; yet is there an expression about it partaking of languor; the sclerotic portion is too white, or has a tinge of blue. The skin may be soft or harsh; in either case there is something of an unpleasant aspect. The circulation is said to be 'languid'—a somewhat indefinite epithet; according to my observation, the pulse is usually more frequent and weaker than usual; the digestive powers are either weak or irregular in their action; the mind may be dull or sprightly, not usually susceptible of long-continued, close application to a single object of study. The impression derived from the general appearance is that there is a deficiency in the powers of the constitution.

Look into the organization of the body, and you perceive all those parts of the body naturally of a red color to be less highly colored than is usual; whilst the white tissues are decidedly augmented in volume. This we hold to be the characteristic of a strumous diathesis, a want of balance in the red and white tissues, the latter predominating. Naturally there is a portion of the white tissues mixed with the rest, and possibly the want of color observed in the last may be owing to the increased mixture of the white. But in many portions of the body there is no admixture of red color; hence they are called white tissues. There is a difference of opinion among physiologists as to the precise manner in which these white tissues are formed and nourished. The opinion that there are two kinds of blood sent out by the heart, one red and the other white—that these two bloods remain mixed until they arrive at the capillaries and then separate, the red to be distributed to the red tissues and then returned to the heart by the veins, the white to be distributed to the white tissues and then returned by the lymphatics, appears to be gaining ground. Supported as this opinion is by Graves and Stokes, of Dublin, by Jackson and Gerhard, of Philadelphia, and many others of the first men in the profession, and plausible and captivating as it is rendered by their expositions, yet I am not prepared to give into it. But as no practical views as to scrofula would be affected by establishing the truth or error of this opinion, I shall not go into an examination of it. Let it suffice to state, that in healthy blood there are found, in due proportions, the elements necessary to form and nourish every texture of the body, and that in every texture there is an apparatus by which these elements are separated and appropriated for such nourishment; that the growth of any organ will be affected, in a great degree, by the amount of such elements brought to it by the blood-vessels, and also by the healthy condition of the apparatus of assimilation.

I do not remember any observations which go to shew any difference in the relative size of the blood-vessels in persons of a lymphatic temperament, as compared with those of other temperaments; but there is evidently a want of red color on the surface of the body, and we might infer a similar state in the interior. Andral and other physiologists have shown that in all alterations of the blood, as one element is increased some others are diminished; indeed, if it had never been demonstrated, common sense would have taught that it could not have been otherwise. The amount of blood, then, remaining about the same, and there being a want of those elements necessary for the nourishment of the red tissues, there must be a superabundance of other elements, amongst which are those necessary to form the white, which are accordingly developed in an inordinate degree.

It will be seen, then, that I understand the state of predisposition to scrofula to consist in an undue predominance of the white tissues, attended and caused by an imperfect sanguification.

*Of Scrofulous Affections.*—I have spoken of some affections as being much more apt to appear in those of a strumous diathesis, and of others as being very much modified by that diathesis. Amongst the first, I would enumerate intestinal worms, pediculi, certain purpura eruptions, particularly of the scalp, enlargements of lymphatic glands, &c. Amongst the latter, the exanthemata, hooping cough, hydrocephalus, inflammations of any part of the body, in which the ordinary results of inflammation of such parts follow, unattended by the formation of tubercular matter. I remark here, that I by no means attempt to give a complete list of either class, and also that many affections not only are more apt to appear in strumous habits, but are also very much modified by that diathesis.

I do not feel competent to settle the long-mooted point, how worms first make their appearance in the intestines, and therefore shall not labor it. We know that they are very apt to appear in children of a lax fibre, of a pale sallow complexion, tumid abdomen, &c. We know, too, that such children are apt to have an undue amount of mucus in the bowels. Now whether we believe that worms are introduced from without, in embryo or otherwise, or that they are formed within by some kind of spontaneous generation, we know that in such cases there is a great aptitude for their fecundation and multiplication, and though we may expel vast numbers of them, they are continually apt to reäppear in equal numbers. We are not so apt to consider pediculi as of spontaneous production, yet we know that in some children it is almost impossible to keep clear of them. In such there is very commonly a dirty, purpuraceous state of the scalp, which we may consider as bearing the same relation to pediculi, as mucus in the intestines does to worms. I am inclined to think that whatever is true as to the production of worms, is also true as to the production of pediculi.

Another affection extremely apt to appear in children of a strumous tendency, is enlargement of the lymphatic glands, particularly those of the neck. I apprehènd these frequently take place without inflammation being present, being caused by acrid matter taken up fròm some scurfy eruption upon the head and carried to them by the lymphatics, causing irritation there.

If I mistake not, M. Lugol dignifies these enlarged glands as '*cervical tubercles.*' I infer this from the general tenor of his remarks, as well as from some special remarks, in which he speaks of their being dissipated in a period which we cannot conceive sufficient to remove tubercular matter. See page 170, op. cit. At other times these enlarged glands are truly inflamed.

The eruptive fevers are all seriously affected by the scrofulous diathesis; measles and small-pox perhaps to a greater degree than the others. It is mainly owing to this diathesis that a very common remark as to measles is so true, viz: that measles is not so dangerous in itself, as it is the source of other fatal diseases, as phthisis, &c. Yet this diathesis renders the treatment of measles itself more difficult, and its termination more doubtful. Hooping cough is another disease often rendered tedious or fatal by a predisposition to scrofula, and, like measles, frequently lays the foundation for phthisis, or some other incurable disease.

But there is perhaps no disease where complication with scrofula is more to be dreaded than syphilis. If attended to promptly and treated with great caution and discretion, the patient may recover without much permanent injury; but if suffered to progress to any extent, or if due caution in the treatment be not observed, there is no telling the amount of wretchedness which may result. Several circumstances conspire to render this complication a source of great mischief. 1st. However flippantly we may talk of the ease with which we can distinguish one disease from another, it is sometimes very difficult to distinguish scrofula from syphilis; so much so indeed, that the highest authorities in our profession have acknowledged that they had confounded them, and treated one when they thought they were treating the other. Even Lugol, who, from his almost exclusive devotion to the study of scrofula, might be supposed to know it well when he saw it, acknowledges his occasional mistakes, and that his diagnosis was only rectified by the effects of medicines. 2d. There is, to a very great extent, an antagonism in the treatment of the two diseases. Thus if a particular symyptom be referred to one disease, and treated accordingly, when in fact it belongs to the other, we not only do no good, but very probably we do great harm. 3d. Although we discriminate correctly and appreciate properly what is due to each disease, yet there is great difficulty in adjusting our treatment so as to bear in due degree upon each.

The scrofulous diathesis, then, appears to modify most other diseases, by rendering them either more suddenly fatal, or else more tedious in their course, more difficult of medication, and more doubtful as to the result. In post-mortem examinations of such cases, little or no difference will be observed from what occurs in such diseases, ending fatally in healthy constitutions.

*Of Scrofula.*—I have defined scrofula to consist in a deposit of tubercular matter in some portion of the body. I have defined tubercular matter to be a pale, yellow or yellowish gray, opaque, unorganized substance. It is true I have seen considerable scrofulous disease, and made a good many dissections of bodies which had died of that disease; but I by no means feel competent to settle various points of its pathology, upon which men, better able to judge and better suitable to observe, differ so widely. I shall therefore briefly state my opinions, and let them pass for no more than opinions. I concur mainly with Dr. Carswell, that tubercular matter is deposited as a morbid secretion, in a state approaching solidity; that inflammation is not essential to its formation, although it many times precedes its production, and conduces to it; that in very many cases inflammation is caused by the presence of tuberculous matter; that there is no disposition or cause in a mass of tubercles to soften, but that that effect is produced by causes extraneous to it; that this softening commences at the circumference, not at the centre.

It is known that M. Lugol thinks tubercles true parasitic animals, produced probably in the same way as acephalocysts. Owing to the great experience and opportunities for observation enjoyed by that great man, his opinion is certainly entitled to great weight. But there are some circumstances attending tubercles which, although not conclusive, are yet sufficiently potent in my opinion to render that explanation improbable. In the first place, acephalocysts do not have a decided disposition to induce inflammation in parts where they are located. Secondly, when inflammation is set up in their immediate vicinity, such inflammation produces no effect upon those parasites. On the contrary, tubercular matter has a strong tendency to produce inflammation, and that inflammation reacts powerfully upon the integrity of the tubercular mass.

Inasmuch as we first recognise tubercular matter in the form of semi-solid points, we are justified in concluding that it is deposited in that form until it shall be detected in a more nascent state.

In addition to observations made by others, which go to show that tubercular matter does appear without an apprecia-tion of previous inflammation, I would observe that I have seen divers instances in which large numbers of small tubercular masses were scattered over the peritoneum, some of which were surrounded by areolæ of distinct inflammation, whilst others had not the slightest appearance of that state. Now, it is clear that if inflammation is essential to the formation of tubercles, it must have subsided before death in the latter mentioned masses. But inflammation is not so apt to appear about very small masses, as about those of some size. But why should tubercles be caused by inflammation exclusively, and yet that inflammation disappear to be subsequently re-produced by the *effect* of its previous action. The theory does not work well. Besides, tubercles are frequently found in situations in which no inflammatory action had ever been suspected, and which exhibited no evidence of its having ex-isted, if the tubercles themselves are excepted.

Although inflammation is not essential to the production of tubercles, yet it frequently precedes that production, nay lays the foundation of that action, upon which their produc-tion depends. We must keep in our minds the difference be-tween concomitants, and cause and effect; also between causes which act occasionally, and those which act universal-ly. I will exemplify my meaning in reference to inflamma-tion by the aid of syphilis. A certain number of persons pre-disposed to scrofula contract syphilis, and afterwards scrofula is developed in them, probably its development is occasioned by the effects produced by the syphilis, which may have been cured, or may still exist. Here we may say truly that syphi-lis is the *immediate* cause of scrofula. But not every man, not even a majority of those who have, or have had syphilis, are afflicted with scrofula. Again, a majority of those affected with scrofula have not had syphilis; then there is no necessary connection between them. Precisely so as regards inflamma tion. It may precede scrofula, may produce a state favorable to the development of it, but it may exist without being followed by scrofula, and scrofula may exist without being preceded or attended by it.

But scrofula frequently gives rise to inflammation. When a tubercular mass has acquired a given size, irritation is set up in the contiguous structures, which is sooner or later followed by inflammation, just as it would take place if any other foreign body were introduced. The object of this process is to expel the offending body. The description given of scrofulous inflammation by Mr. John Burns, (but what I have proposed to call *tubercular* inflammation,) appears to set this matter in the proper light. 'Scrofulous inflammation, says he, is marked by a soft swelling of the affected part, which very frequently is one of the lymphatic glands. The covering or coat of the gland becomes slightly thickened, and its substance more porous and doughy; the swelling increases and the doughy feel changes by degrees into that of elasticity or fluctuation, and a firm circumscribed, hardened margin can be felt round the base of the tumor. If at this time an incision or puncture be made, either no matter or very little is evacuated; the lips of the wound inflame and open, displaying a sloughy looking substance within; and between this and the skin a probe can often be introduced for some way all around. If, however, the disease should have advanced further, there is very little elasticity in the tumor; it is quite soft, rather flaccid, and fluctuates freely; the skin becomes of a light purple color, and small veins may be seen ramifying on its surface. Sometime after these appearances, the skin becomes thinner at a particular part, and here also it is generally rendered of a darker color. It afterwards bursts, and discharges a thin fluid, like whey, mixed with a curdy matter or thick white flocculi.' This description explains satisfactorily several points of the pathology. 1st. The tubercular mass, being a foreign body, causes irritation and inflammation for the purpose of expelling such body. That inflammation, of course, begins tathe surface of the body to be expelled. A fluctuation occasioned by the presence of pus around the mass is felt, and the hard edges marking the bounds of suppuration are evident. But if an incision be now made, very little pus is discharged; but there is a sloughy looking substance within, between which and the skin a probe can be introduced for a considerable distance. Why this? Because although little matter has been found, yet that little is spread all over the surface of the tubercular mass; therefore a probe can be passed freely round it. But when the disease is farther advanced, when the surrounding parts have had time to pour out fluid sufficient to soften the whole mass, then fluctuation is free, and the pus when discharged is mixed with softened tubercular matter.

Laennec taught that tubercles commenced softening in the centre; but as his observations were made upon the lungs, his facts and observations admitted of two explanations, either of which *might* be true; to-wit: upon making a section of the lung, various branches of the bronchial tubes and air cells would be divided; some of these might contain solid tubercular matter, others might contain a considerable portion of the same matter, with the centre containing more or less fluid. His explanation was that the whole had been solid, but the tubercles had begun to soften in the centre. Dr. Carswell explains it differently; that the deposit of tubercular matter commences on the mucous surface of the vessels and air cells; that, as fresh portions are deposited, that first excreted is forced nearer to the centre, until the vessel or air cell, as the case may be, is completely filled; that when this cavity in the centre is observed, it is because the cavity has not yet been filled; that instead of being in a more advanced state than those which are filled with solid tubercles, it is in truth less advanced. This appears to me the more rational solution, the more especially as tubercular masses found in the brain and cellular substance do not show this disposition to soften in the centre. It is not denied that occasionally spaces may be found in large tubercular masses; but these depend upon deposits beginning at various points and approaching each other by continued aggregation.

There is no structure of the body in which tubercles may not be deposited. The mucous membrane lining the air vessels, the parenchyma of the lungs, the mucuos and serous membranes generally, the cellular tissue, the brain and nerves, the bones, any or all, may be invaded by them.

*Treatment during the state of predisposition.*—We are not very apt to be consulted during this state, unless in families in which several of the members have already fallen victims to some form of the disease. Then there will probably be a very decided tendency to the development of the complaint. I doubt the propriety of giving much medicine during this state; but cases may occur in which more or less will be required. But much can be done to ward off the disease by due observance of hygienic rules. Two things are of paramount importance. 1st. To keep the digestive organs in a state of health. 2d. To supply them with a due quantity of digestible nourishing, not stimulating food. One very potent means of keeping the digestive organs in a healthy state, consists of a due amount of exercise taken daily in the open air, in the blessed light of heaven. It will not do that a given amount be taken every week or month; it should be taken every day, at least every day which ordinary prudence will permit going out; not less than three or four hours daily of such exercise as can be borne without fatigue. It may be said, and there may seem to be reason in it, that the patient is unable to bear the desired amount of exercise; but muchof this depends on a sufficient effort not being made. Many persons can go abroad very well, who are fatigued in returning home. We can all bear more fatigue when in pursuit of anything that interests or amuses us, than when we have no object in view. We should, therefore, give our patient every chance to profit by his exercise. It should be made to interest and amuse him. Let him labor moderately at any handicraft for which he may have a taste; work in a garden; engage in botanical or geological explorations in his neighborhood; hunt game; anything, which will cause him to spend a considerable portion of his time upon his feet and in the open air. This will invigorate his muscular system, his circulatory system, and indeed all the organs of the body. Let him by no means forget that the light of the sun is a powerful agent in the preservation of health. Most persons are fully aware that the light of the sun is indispensable to the health of vegetables, but many seem to think that animals can do very well without it. I will here relate a case, the parallel of which may be found almost any day when search shall be made. Some years ago, I had a child born during the fall. My wife continued very much indisposed during the winter; so much so, as to keep the house. Our family room was quite dark. In consequence of the child being confined so long without exposure to light, he was very fair and delicate in his appearance, yet sufficiently plump and lively, indeed having every appearance of good health, except a suitable complexion. Having noticed this, I caused him to be taken into the open air every day when the weather would by any means admit. His complexion soon began to improve, and in a month or two he had as rosy a face as there is any need of. Butchers, too, are aware that animals when fattened in dark places furnish a meat much fairer than when fattened in the light. Light, then, is as necessary to the health and perfection of animals as it is to those of vegetables. In his labor or exercise, let him avoid exposure to cold damp air whilst he is in a state of inactivity, or insufficiently covered; especially let him avoid getting his feet damp and cold whilst inactive. A man might wade in water for hours without inconvenience, provided he dry and warm his feet when he quits, whereas one-fourth or one-tenth of the time spent standing upon damp cold ground may give an attack of colic, of pleurisy, or some other serious disease. In the first instance, the warmth and vigor imparted to the body by the exercise of walking enables it to ward off the influence of cold, but in the latter case there is no such prophylactic. Let him sleep in a dry, large chamber of suitable temperature. Excessive cold, say from 15° or 20° downwards, should be avoided, especially during sleep; but even that temperature may be safely borne when due exercise is taken. Let his mind be kept in activity, but not harrassed long at any one time, and as pleasantly occupied as circumstances will admit. If the family has suffered from scrofula, or indeed from any single disease, his mind should not be allowed to dwell upon such occurrences. All possible means should be resorted to to divert his thoughts from cases of a fatal character, which may have occurred in the family. He should never hear any remark as to his resemblance to any member of the family who may have died. He should never be permitted to talk about the ill health of the family. On the contrary, he should be encouraged to engage in pleasant enlivening conversation. Some forty years ago, it was usual for young people of both sexes to romp a good deal. It very rarely, if ever happened, that this was carried to such excess as to be liable to cause severe cold, as is too frequently the case after dancing. It was more enlivening and less dangerous than this latter; and really I do not know that it was less genteel than waltzing. Gentility, however, has put its ban on romping, without furnishing us with anything so good, at least so well adapted to preserve health in the present state of society.

Nothing perhaps is of more importance in the preservation of health, than a due regulation of the bowels. Nearly every function of the body is to be performed at stated intervals. At such intervals there is almost always a distinct prompting on the part of nature, if not a full performance. The intervals between the times for expelling the feces appears to have been fixed by nature at twenty-four hours. So true is this, that it may be said to be universal; and it is extremely uncommon that there is any exception depending on nature. Every man, who has not by his own neglect or mismanagement broken up this circle of action, feels at a given hour every day an inclination to perform that act; and so long as he attends to the promptings of nature, so long his bowels will remain regular. Then it is a matter of great importance that all young persons should be impressed with the absolute necessity of attending to these promptings, and the preservation of this regularity. This is the more especially true as regards those who have any tendency to delicacy of health. All the functions of the body are more or less intimately associated; the derangement of one having a tendency to derange some other; and the preservation or re-establishment of one having a like power to preserve or re-establish the healthy action in some one or more. But there are exceptions to the universality of this law, perhaps, however, depending on some degree of disease. If disease can be detected, that must be treated according to its nature; but if no disease can be ascertained, we must address ourselves to remove the irregularity.

We will suppose it is a child, who is troubled with costiveness. It will many times be sufficient to rub the hand gently over the abdomen for ten or fifteen minutes, three or four times a day. If that should not suffice, then an injection of warm water, administered daily, at a given hour, will answer. After a few days the injection may be delayed somewhat beyond the usual hour, (but the frictions continued,) to ascertain the impression made upon the habit. If necessary, the same treatment may be resumed. These means alone, indeed the frictions upon the abdomen alone, will very frequently suffice; and I hold it a matter of great moment to restore healthy action in the organs by treatment purturbating in the least possible degree. If the patient be of age sufficient to understand directions, he should be taught to perform these abdominal frictions for himself, and then to retire at a stated hour every day, and endeavor to procure a motion from his bowels. He must be impressed with the great importance of his perseverance and success to his subsequent health. It must be confessed that it is very difficult to make children and young persons appreciate the vast importance of success; but on the other hand, as the habit of costiveness is not usually inveterate in such patients, less time is necessary to secure success. From much experience, I am fully satisfied that this alone is sufficient to break up any habit of costiveness, which does not depend upon disease. I have never known a failure where a fair trial had been made.

Besides securing a healthy condition of the chylopoietic organs, we must see that they are supplied with a due quantity of suitable food. There is something peculiar about many stomachs which will enable them to digest particular articles of food, which are usually found difficult of digestion; or, on the other hand, incapacitates them from managing articles which are usually considered easily digested. Such peculiarities ought always to be ascertained and attended to. Anything which disagrees with the stomach is to be avoided, no matter how easily it may be digested by others; and occasionally an article, which to most stomachs is highly indigestible, may be taken without injury, or even with advantage. Bearing these remarks in view, most of our vegetables, milk, eggs boiled so that the whites only are done, nearly all flesh usually eaten as food may be taken in moderation. All flesh should be cooked so as to be juicy, and not highly seasoned; none that is dry or crisped should be touched. Sausages and all high-seasoned gravies should be prohibited without qualification. Minced pies, and indeed pastries in general, should also be under ban, rice and bread puddings being excepted.

Important to health as is a proper condition of the chylopoietic viscera, that of the skin is no less so. Hence great attention must be paid to it. Flannel should be worn next to it at all seasons. In summer this may be thought oppressive, or at least not necessary. But nothing is more effectual in preserving the skin in a healthy, perspirable condition. Although it may be somewhat unpleasant in very hot weather, yet that is precisely the kind of weather in which free perspiration is liable to occur, and when it has taken place much injury is frequently done by a sudden impression of cold. The clothing being separated from the body for a short time, becomes sensibly cooled, and upon reapplication, if it is of materials which conduct heat readily, communicates a sensation decidedly chilly, and that to a degree sometimes sufficient to produce disease. But the flannel protects the body not only against sudden vicissitudes of weather, but also against such occurrences as that just mentioned. The surface of the body should be kept clean. Bathing should be used two or three times a week, or even every day. Scarcely any one who is not diseased will be injured by the cold bath when prudently used. A cold bath should never be used when the body is fatigued by long-continued exercise. In cases now under consideration, it is doubtful whether it should be used at any time when there is free perspiration or the body much heated. The danger will consist in the suddenness of the shock, and dubiety of reaction. As a general rule, the application of the water should be very short, as in the shower or plunging bath; after such, the reaction is much more apt to take place. It is thought, too, that salt baths are less liable, *ceteris paribus*, to be followed by imperfect reaction. One general remark should always be held in remembrance, viz: if, after the bath, a pleasant sensation of warmth ensues, benefit may be expected from it: on the contrary, if the body feels chilled and uncomfortable, no good, but injury, may be expected from a continuance of the practice.

Daily friction of the surface is another important means of preserving it in a healthy condition. By friction, much of the old dry cuticle is removed, and thereby a free elimination of the insensible perspiration allowed. The vessels of the skin also are thereby stimulated to healthy action.

It only remains that I say a few words as to climate. We shall generally have to treat the disease where we find it; yet all places are not equally favorable to the successful treatment of this disease. Hence when circumstances admit of change of location, it may occasionally be made with great advantage. As a general rule, a situation having a dry, salubrious air is to be preferred. Some observations recently made would seem to indicate that a location where intermittent fever is common, would be advisable for phthisical patients; and if so, it would appear probable that other varieties of scrofula would also be benefitted by the same location. This would seem opposed to what is pretty well ascertained as to scrofula. One or the other must be wrong. Nature never teaches one thing and philosophy the opposite. I am therefore inclined to think that the observations referred to have been hastily and imperfectly made, and therefore will be set aside when more extensively and thoroughly tested. Scrofulous persons do not generally bear cold very well, therefore removing a few degrees south, taking care to secure a dry and healthy location, may be safely advised. Change of climate, like mercury, may be the means of doing much good when used with discrimination, and therefore within due bounds; but will almost certainly do much harm when carried to excess. Damp locations must be avoided. It is well known that both damp cold air and damp warm air, are much more oppressive than dry air at the same temperature.

From what has just been stated, it would appear that because a location in Florida benefits a patient from Virginia, it does not follow that it would be equally beneficial to one from Maine or Canada; or that the Virginian would have been more, or as much benefitted by a similar location in the West Indies. To secure the best result from change of climate we must judge of the capabiliιy of each patient to bear that change, and to be benefitted by the air of the place to which we send him. Some will require a greater chιnge than others. I think less discrimination has been exercised on this point than is desirable.

*Treatment of Scrofulous Affections.*—It will most frequently happen that we will not be consulted until the period of predisposition has passed away, and some of the thousand affections which go under the name of scrofula have made their appearance. Although scrofula properly so called, at least such as I have defined it, may not be present, yet it is evidently approaching. This is the time to do good, if ever. To do good, it is necessary to take a very patient and scrutinizing view of our patient's condition. In common language he has scrofula, but cases of scrofula may differ as widely as cases of fever. Here we find the great difficulty in arriving at just conclusions. Scrofula has been considered a *spccific*

*disease*, and the profession have been looking out for a *specific remedy;* and in pursuance of that object, have thrown common sense overboard, and put themselves under the influence '*of old wives' fables.*' Hence the vacillation in the estimation of divers remedies at various times. One patient was benefitted, possibly cured by mercury, and forthwith mercury was the catholicon. Soon after, another was injured, or at least got worse during the use of this remedy, therefore it was anathematized. Anon *cura famis* was all the rage. If indeed it had turned out *cura* it had been well. But alas! the disease proved no more amenable to *fames* than to *mercury.* It were bootless even to enumerate all the *humbugs* (to use a common political term,) played off upon the profession from the days of mercury down to those of *iodine* and *walnut leaves.* To my mind it appears pretty clear that much of the reputation which each article in succession enjoyed, was due to the imagination of the man who employed it. And yet I doubt not that many of these panaceæ did in truth do good and cure patients, *when the disease was adapted to the remedy;* for I doubt very much whether the time has yet arrived when the *remedy is adapted to the disease.*

It would be endless to take up all the varieties of scrofulous affections in detail; I shall therefore mention only a few. I will suppose a case, in which there is considerable swelling of the cervical glands, attended by bloated face, thickened upper lip and of the '*columna alaeque nasi*' and tumid abdomen. These cervical glands may be inflamed, or they may be only irritated by acrid matter taken up from the scalp or some adjacent part. It is evident, however, that unless something is done to improve the health, serious disease will ensue. A little examination will shew us that the digestive powers are deranged, and that sanguification is imperfectly performed. Mucus or worms, or both, may be oppressing the bowels, or they may be costive, the liver acting imperfectly; or there may be gastric derangement, causing a secretion of tough glairy phlegm, or of salt or acrid matter, often attended by the presence of tough phlegm about the larynx, which occasions a disposition to cough frequently, and as it is separated with difficulty, retching is often produced. These secretions also frequently produce flatulence of the stomach, cardialgia, and tenderness of the epigastrium. In any of these events, some purging is necessary, and mercury in some form should be an ingredient, used more or less frequently for that purpose. I have enumerated mercury as one of the prominent causes of the development of scrofula. Some may think, therefore, that there is an inconsistency in recommending it as a remedy; and I will not deny that there may be extreme cases in which, owing to idiosyncrasy, no amount of mercury can be taken without danger. Indeed we ought always to be on the look out for such cases among scrofulous patients, because of the great injury which may result from irregular or excessive action of mercury. But I speak of the overwhelming majority of such cases. Lawyers have a maxim 'that the abuse of anything is no argument against its proper use.' The principle is as true in medicine as it is in law. The purgatives should be repeated at suitable intervals, until the stomach and bowels shall be relieved of worms or mucus; and until they, together with the other digestive organs, become in a healthy condition. We shall many times receive much advantage by premising two or three emetics, or by alternating them with the purgatives. As an emetic in such cases I prefer ipecac to tartar emetic. This course alone will sometimes suffice, when the diathesis is weak and application is made early. And such, I apprehend, has been the cause of the credit that mercury and emetics have respectively enjoyed at different times in the cure of scrofula.

Here, I will say a word as to the employment of emetics and cathartics. A number of physicians, respectable both as to number and talents, depend almost exclusively upon emetics as a remedy for scrofula. Of these, one portion give them at shorter or longer intervals, almost indefinitely, or at least till the disease gives way, or else they arrive at the conclusion that the disease is incurable. The result of this course is sometimes very fortunate, especially where the case is not a severe one, and the stomach recovers pretty well from one emetic to the other. If, however, the disease be grave, and do not yield readily to emetics, and they be repeated at too short intervals, considerable irritability of the stomach may be induced, which will add greatly to the trouble in the subsequent management of the disease. Another portion of those who rely exclusively upon emetics, give them until they produce '*yellow, green or blue bile*,' that appearing to be the object of their administration. My own belief is, that when carried to the extent of producing *green* or *blue* bile they have been pushed too far. I do not say that when green or blue bile is produced by vomiting, such emetic was improper. I know such is not necessarily the case. Such bile is an evidence of irritation, or some unhealthy action in the liver or primæ viæ, and an emetic may discharge from the stomach the offending cause; or it may communicate an impression to the liver which shall tend to correct that irritation there. What I maintain is, that when this result takes place as the *effect of vomiting* in such cases, we should not look upon it as a fortunate result, but the contrary. Therefore I would in such cases, so far from being encouraged to persevere in their use, feel myself called upon to suspend them, and use means to remove the irritation which I had caused.

We are directed, too, to use purgatives until the feces become of a healthy character. But it unfortunately happens that purgatives, like emetics, are not free from danger in their indiscriminate employment. 'Stools of considerable consistence and of a dark green color' are considered as a favorable result of mercurial cathartics. This is true, provided the 'dark green' is of the *right color*, and the stools composed of bile. But this description of such stools will not secure the young physician against an error of momentous consequence. I have seen physicians of considerable age and experience persevering in the use of mercurial purgatives, when there was a great quantity of dark green *mucus* discharged, (the color being something darker than that of Jamestown weeds and of a shining appearance,) wondering all the time that the patient should continue to grow worse under 'such good mercurial purging.' This kind of purging I hold to be caused by irritation. It takes place when no calomel is given, to be sure, but is likewise frequently produced by it. In the employment of mercury in this disease, we must avoid two things, too much irritation of the bowels, and the constitutional effects of mercury. It is only when such effects follow its use, more especially the latter, that it acts deleteriously in scrofulous habits.

If the disease be somewhat grave, it will not yield to simply putting the digestive apparatus in good order. It will be necessary to keep it so for a considerable period, and in the mean time correct and invigorate various other functions. To assist us in this, we shall need, among other things, tonics. Various articles of this class have been, at different times, esteemed, if not as specifics in this disease, very nearly approaching that character. Although we may reasonably believe that articles of this class are occasionally of much benefit, it would be difficult to point out any peculiar qualities in any one article, which would indicate its right to be considered greatly above others as a remedy entitled to a monopoly. Bark and iron are perhaps more to be relied on than others, unless indeed we put iodine in the list. Bark was at one time very popular, but like other articles, being taxed above its ability, it fell into disrepute because it could not accomplish imposibilities. Yet is it a valuable tonic in such cases as have been duly prepared for its use, by properly correcting the condition of the digestive organs. It, or rather quinine, should be given in moderate doses, say two grains for an adult three times a day, an hour before each meal, and continued for a considerable time. An occasional emetic or cathartic should be interposed, as may be indicated by the condition of the stomach and bowels. Iron is a tonic which appears well suited to many cases of scrofulous affections. In cases in which worms or mucus have loaded the bowels, and have to a considerable extent been evacuated by purgatives, iron is particularly well suited to strengthen the bowels, and prevent the reaccumulation of such sources of oppression. Indeed, of itself it will frequently expel the worms. Hence, with some physicians, it is highly esteemed a vermifuge. As there is most commonly an imperfect sanguification, iron, by furnishing an ingredient necessary to the blood, may be considered as an article of diet, as well as a tonic. Of the preparations of iron, we should choose one, the dose of which is small, which possesses considerable activity, which keeps well, which is not apt to oppress the stomach, and upon which the stomach acts readily. These qualities are possessed in a high degree by the *Pilulæ Ferri Carbonatis, U. S. P.*, or *Vallett's Ferruginous Pills.* Given in the quantity of 10 to 30 grains daily, they exert a most invigorating effect upon the health, increasing the amount of coloring matter in the blood, they favor the development of the red tissues, and of course improve the complexion. It is true that Dr. Elliston, of London, has used, with much benefit, the *Rubigo Ferri* in doses of from ʒii. to ℥i., but it does appear to me that this must be a very disgusting mode of using a good article, and one very much calculated to oppress the stomach. I think that when medicine is to be taken for a long time, it is very important that it be rendered as little disagreeable as possible. I believe that unpleasant sensations occasioned by the administration of medicines, are a great drawback upon the ability of the stomach to act upon them beneficially. After all, the benefits resulting from medicine depend, not so much upon the quantity taken, as upon the mutual action and reaction of it and the stomach on each other. When in the course of treatment it becomes necessary to use an eccoprotic, I have given with much satisfaction a pill composed of *Sulph. Ferri Hiera*, *picra*, each ℥ss., *jalap*, *myrrh*, each ʒi., made into pills of common size, of which two or three may be taken at night. Whilst these pills co-operate in the general plan of treatment, they appear to me to possess one very important quality, that instead of requiring the dose to be increased after being some time used, it can be gradually diminished, and at length altogether dispensed with. Great diversity exists both as to the usefulness of iodine in scrofulous complaints and as to its mode of action. I am disposed to place it among the tonics,* *when used in such a manner as to be serviceable.* Hence Lugol and some others declare that during its administration the appetite improves and the body increases in weight. But we are assured by others that it produces irritation of the stomach and emaciation of the body. I apprehend that at this day there is no difficulty in believing that any article may be so managed by different persons as to produce dissimilar or even opposite effects. I believe that much of the discrepancy of testimony as to the effects of iodine, is due to the different modes in which it is used. I can well believe that, used in the quantity of a grain a day, it may act as a tonic, and in the quantity of five or six grains daily it may act as an irritant. To set this matter in a proper light, I have thought it worth some trouble to collate a number of formulæ for the administration of iodine, and to calculate the quantity of that article given for a dose according to each formula. I shall premise ʒi. of a watery solution dropped from a two ounce vial gives about sixty drops, of an alcoholic tincture about 140 drops, of an ethereal tincture about 180 drops. Varieties to a considerable extent will take place, occasioned by the purity of

*NOTE.—Some physicians think that the tonic effects of iodine are exerted principally or wholly on the absorbents, thus enabling them to take up the morbid deposit. Others say, that this effect is produced by its action as an *alterative*, the action of the absorbents being not only increased but altered. Others again consider it as a *deobstruent*. I certainly think an appropriate name a very important thing; but unfortunately in medicine, as in other departments of philosophy, men will sometimes express the same idea by different words, and sometimes different ideas by the same words. It is very important, then, that we understand correctly the idea intended to be conveyed. My idea then is, that administered in suitable doses and under appropriate circumstances, iodine increases the appetite and promotes digestion, furnishing a more healthy chyle; that, in consequence, the various organs of the body are enabled to take on a more healthy action; that the nutrient vessels being furnished with a better material for assimilation, are also enabled better to select the most appropriate molecules. As a consequence of the restoration of healthy action, any effete or deleterious corpuscles, deposited in the texture, are taken up and removed. In this way it may be held to act both as an alterative and a deobstruent; but I conceive that its whole action may be referred to its tonic powers.

the alcohol and ether, also by the size of the rim around the mouth of the vial, and especially by the fluid running back so as to touch the neck of the vial before it drops.

No. 1. Pills of iodine. ℞ iodini, $\frac{1}{8}$ to 1 gr. twice a day. (Appendix to Lugol on Scrofula.)

No. 2. ℞ iodini, gr. ss. (Pereira.)

No. 3. *Tincture of Iodine.* ℞ iodini ℥i., rectified spirit f℥xvi., m. v. to fʒss., gr. $\frac{1}{4}$ to $1\frac{5}{8}$. (E. and U. S. P.)

No. 4. ℞ iodini ℈ii., S. V. R. ℥i., gtt.iv. x. xx. ter. die, gr. $\frac{1}{7}$, $\frac{5}{4}$, $\frac{5}{7}$. (D. P. and Magendie.)

No. 5. ℞ iodini ℈ii., S. V. R., ℥i. spt. lavend. ʒii., gtt. 10, 20, 40 thrice a day, gr. $\frac{2}{7}$, $\frac{4}{7}$, $1\frac{1}{7}$. (Ellis' Formulary.)

No. 6. *Ethereal Tincture.* ℞ iodini, gr. vi., ether. sulph. ʒi., dose, gtt.x. bis die, gr. $\frac{1}{3}$. Ellis says 30 drops of this tincture contain gr. i. iodine. Appendix to Lugol says, 10 drops contain gr. i. iodine. Ellis is right, whilst the appendix has mistaken drops for minims.

No. 7. *Compound Tincture Iodine.* ℞ iodini, ℥i., iod. potass. ℥ii. S. V. R., Oij., mfx. to fʒi., gr. $\frac{1}{3}$ to 2. (L. and U. S. P.) Ellis directs gtt.v. xv., pro re nata, equal to gr. $\frac{1}{15}$ to $\frac{1}{5}$.

No. 8. *Solutions of Iodine.* ℞ iodini, gr. v., iod. potass. gr. x., aq. destill., Oi., fʒii. to fʒvi., gr. $\frac{1}{12}$, $\frac{1}{4}$., (L. P.) To this formula, appendix to Lugol adds: 'Doses x. xx. bis terve in die.' If this x. xx. refers to drops, as I presume it does, the dose will be $\frac{1}{154}$, $\frac{1}{77}$ gr.

No. 9. ℞ iodini, ʒvi. iod. potass. ℥iss., aq. destill. Oi. gtt. xx., gr. $\frac{9}{10}$. U. S. P.

No. 10. ℞ iodini, gr. iii., potass. iod. gr. vi., aq. destill. ℥i., gtt.vi. x. ter. die, gr. $\frac{1}{27}$, $\frac{1}{16}$. Ellis' Formulary.—gtt.iii. v. gr. $\frac{1}{54}$, $\frac{1}{32}$. Morton.

No. 11. ℞ tincture iodine fʒi., mucil g. acaciæ f℥ii., aq. destil. f℥vi., ℥ss. 2d q. hora, gr. $\frac{1}{4}$. Ellis.

No. 12. ℞ iodini ℈i., iod. potass. ℈ii., aq. destill. ℥vii., gtt.

vii. bis die, gr.¼. Ellis. I presume this is a mistake, and intended as a transcript of the following.

No. 13. ℞ iodini, ℈i., potass. iod. ℈ii., aq. destill. ℥viii., gtt. vi. bis die, gr.¼. (Lugol.) Each week the dose is to be increased 2 drops until it reaches 30 or 36, maximum gr. 1½.

No. 14. ℞ iodini gr.ss., potass. iod. ʒss., syr. papav. ℥ss.. aq. destill. ℔ss., ℥ii. ter die, gr. ⅛. Dunglison, N. R.

No. 15. ℞ iodini gr.ii., iod. potass. ʒiv., aq. menth. destill. ℥vi., ℥ss. ter die, gr.⅙. (Magendie.)

No. 16. ℞ iod. potass. ʒss., aq. destill. ʒi. solve, et adde iodini gr.x., gtt.v. xv. daily, gr.⅝ 2½. (Append. Lugol.)

*Preparations of Iodide of Potassium.* No. 1. ℞ iod. potass. gr.ii. sulph. magnes. ℥ss., tart. emetic gr.ss., aq. destill. ℥vi., ʒi. 3 4 ve. die, gr. 1/24 iod. pot. Ellis.

No. 2. ℞ iod. potass. gr.xxxvi., aq. destill. ℥i. gtt.v. xx. ter. die, gr.⅛ 1½ iodide. Magendie.

No. 3. ℞ hydriod. potass. ʒss., aq. destill. ℥i., gtt.xx. xxx., to ℥i. daily, gr.1¼, 1⅞, ʒss.

No. 4. ℞ iod. potass. ʒiiss., aq. destill. ʒiii., micae panis q. s. pill no. cl. divid. no. ii. ter. die., gr.ii. (Appendix Lugol.)

It will be observed that in the foregoing estimate of the preparations of iodine, I have not taken into consideration that which is present as an ingredient of the iodide of potassium. It appears that iodine is very much modified by combination with other articles, as it can be used in much greater quantities when thus combined than in its pure state. Thus it is very irritating when used in the form of a pill or tincture, because of its local action. Indeed, I do not think it ought ever to be used in either form. By being dissolved, by the aid of iodide of potassium, it is much more manageable; by being combined with potassium, iron, &c., it can be taken in much larger quantities, so much so, that Lugol considers the iodide of potassium simply as a solvent for iodine, although there is half as much more iodine in the solvent as is dissolved in it.

Not being qualified to state the relative value of the two, I have gone upon the supposition that Lugol is right in considering the iodine as of little activity.

It may be remarked that although the 10th formula directs only from $\frac{1}{27}$ to $\frac{1}{16}$ or $\frac{1}{52}$, $\frac{1}{54}$ of a grain of iodine thrice a day, yet Drs. Morton* and Ellis direct us to diminish the quantity if it 'produce dizziness, pain in the bowels, or other unpleasant symptoms.' If caution is necessary in using that preparation, it would seem much more called for in a large majority of them. No directions are appended to some of the formulæ, so that we cannot say what amount is intended to be taken daily; but we may presume, when not otherwise directed, thrice daily. By this calculation (leaving out of the account the latter directions appended to the 8th formula as ambiguous,) we have the daily quantity from $1\frac{1}{8}$ (10th formula) to 6 grains (7th formula). I believe, as indeed is believed by others, that Lugol has been more successful in the employment of this article than others, because he has used it in moderate doses, and has paid great attention to other means of improving the health, as exercise, clothing, diet, &c. Many physicians, both of Europe and America, declare most unequivocally that they have never seen it do good. As to the mode in which they used it, as well as the suitableness of the cases for its use, we know little. One thing, however, I do know, viz: that men eminent in the profession have their prejudices and caprices as well as their more humble brethren. And when they have been foiled, in consequence of having used an article in a case not adapted to its employment, they are no less apt to denounce the remedy. In the successful employment of any remedy, much depends upon its being properly prepared, and many medicines have fallen into unmerited neglect from a want of attention to this circumstance. I apprehend that this is as emphatically true of iodine as of any remedy under the sun. I have seen a gentleman occupying an exalted station in the profession who used the tincture of iodine freely, and kept it as an *officinal preparation.* Now I

---

*Illustrations pul. consumption, p. 130.

apprehend that this is the most objectionable mode of administering the article. At least I have never given it without producing irritation of the stomach and vomiting, occasioned, doubtless, by the decomposition of the tincture by coming in contact with water. I presume the gentleman above mentioned escaped producing much irritation, by having his tincture pretty well decomposed by age before it was administered. But what reliance is to be placed upon experience of this character? If we employ it, let it be in some form in which it is not liable to be set free, and thus produce disorder of the stomach, as a solution with hydriodate of potash.

A combination of these last two articles, namely, iodide of iron, would seem to promise much in a disease in which each is so valuable. I apprehend, however, that the proportion of iron is too small for us to expect much from that. When we wish to employ the two articles in the same case, I think we had better use a solution of iodine, and give Vallet's ferruginous pill in addition. We can thus give just as much of each article as we please. I have employed the article, and apparently with benefit, though I do not know that any better result attended the administration than would have attended the use of iodine. No article in the materia medica requires to be prescribed more carefully than this; and no physician ought to administer it without knowing its purity. He cannot send to an apothecary for it with any assurance of getting what he wants, at least such has been my experience. I have never seen the article that did not contain sesqui-oxide and sesqui-iodide. The sesqui-iodide is greatly more active, and when given under the impression that we are giving the iodide, never fails to irritate the stomach. The syrup of iodide of iron is said to keep well, yet would I be afraid of that if not fresh. Perhaps the least objectionable formula for this article is that of pills as recommended by Christison, to-wit: R. iodine 127 grains, clean soft iron wire ℥ss., distilled water 75 minims; agitate briskly in a strong vial with a glass stopper until the froth becomes white; pour the liquid upon two drachms of finely powdered loaf sugar in a mortar; triturate briskly for a few minutes; add gradually a powder composed of liquorice powder ℥ss., pulv. gum arabic ʒiss., flour ʒi. mix., divide into 154 pills. Each pill contains one grain of the iodide. During agitation this preparation evolves a very considerable amount of heat, so that it is necessary to envelope the vial in a cloth. So much gas is evolved also as to require great care that the stopper be not displaced and a portion of the ingredients lost.

Whilst we thus attend to improving the general health, we should also have an eye to the local affection. To remove it, volatile liniment will frequently be sufficient. If somewhat obstinate, some of preparations of iodine may be used topically. Hydriodate of potash in the proportion of ℈i. to ℥i. of nice lard. If a more stimulating article is needed, from 3 to 10 grains of iodine may be added.

It will be seen that it will not do to rely upon emetics, cathartics or tonics exclusively; neither can we depend upon them altogether, however judiciously combined. We must call to our aid means more properly hygienic than medicinal. In the first place, I will mention bathing. Cleanliness must of course be insisted on, but bathing, properly used, is a very important means of improving and preserving health. Scarcely any circumstances preclude the occasional use of the warm bath. It removes slight irritations, and produces a train of pleasurable sensations highly conducive to health. A moderate quantity of salt added to the bath will make it more effectual in stimulating the skin; or the surface may be rubbed dry with a towel which had previously been wrung out of salt water and dried. If we wish to make the bath medicinal, we may use a solution of iodine as recommended by Lugol. Cold bathing is less universally applicable, and should always be commenced with caution. Rules by which we may judge of its usefulness and safety have been already mentioned. If it can be used safely, it will almost certainly be beneficial. What has been said as to the means of preserving health under the head of predisposition, applies with much increased force here.

Having gone so fully into the mode proper in the treatment of enlarged lymphatic glands, which applies very generally to what is usually denominated the different forms of scrofula, I will dismiss this branch of the subject with some general observations. 1st. All affections, whether considered scrofulous or not, occurring in persons of a strumous diathesis, bear depletion worse and tonics better than the same affections occurring in healthy constitutions. 2d. Parts affected with inflammation bear exercise better; thus in scrofulous ophthalmia it is not so necessary to exclude the light as in common ophthalmia, nay, a certain amount of light is beneficial. Again, scrofulous white swelling, after the acute stage is over, is benefitted by exercising the limb moderately. I have seen a case in which the tibia was denuded for 10 or 12 inches in length, and over full four-fifths of its circumference improve with great rapidity whilst the patient was permitted, after some constitutional treatment suited to the first stage, to walk upon it daily. 3d. That during our treatment inflammation of some part may be set up. Whilst this continues, we must intermit our tonic course until, by appropriate means, we shall have subdued this inflammation, and then resume it. 4th. That whilst we attend to constitutional treatment, we must not fail to make such topical applications as may be suited to each particular case. 5th. That much time is necessary to manage these cases successfully. We cannot, if we would, make a great revolution in the constitution in a short time; and if we could, we should probably produce an evil equal to the one we cure. A neglect of this consideration has been the means of much injury to patients, and of much discredit to remedies. We try to do too much in a short time. As a friend of mine thought, for whom Fowler's solution of arsenic had been prescribed for intermittent fever. As the next paroxysm was very much mitigated, he said, 'if a little is good, a good deal is better,' so took half an ounce. 6th. It is confessedly very difficult to appreciate correctly the effects of remedies in this disease. There is not so much danger in misconceiving the immediate effects of our remedies, when given in decided doses, but when they are given in small doses for a long time, the case is very different. I think I have seen the effects of such treatment appear weeks after it had been discontinued; at least, if the amendment was not occasioned by that treatment, it was the effect of the powers of nature, for no other remedies had been employed. It is easy to conceive how the employment of another medicine, about this time, might give rise to very erroneous opinions as to the effects of each. 7th. To all of the exclusive modes of treatment, I would apply one remark. That so far as they are exclusive, they are wrong; yet when used with discrimination, and under proper circumstances, each one may be made very useful. Even the *cura famis*, when used to a certain degree and continued for a short time, may be the means of strengthening the digestive powers, and therefore beneficial. But if carried too far, it proves hurtful. And I think it quite probable that much of the credit which this mode enjoyed was acquired by the inability of the patient to pursue the treatment to the degree prescribed, and his unwillingness to inform his physician of the fact. A case directly in point came to my knowledge a short time since. An eminent member of the profession thought himself entitled to some credit in consequence of a cure effected in this way. The patient, however, put a different construction upon the affair. He said he stood the low diet as long as he could, but gave it up. 'Tis true he took the soup and gruel as prescribed, but he took the beef and potatoes, bread and milk likewise.

*Treatment of Scrofula.*—Although we cannot expect to cure scrofula often, yet we shall occasionally meet with cases in which the diathesis is weak, and the accumulation of tubercular matter circumscribed, in which we may hope to witness a restoration to health. Whether that restoration be a *cure* or a *recovery* is perhaps doubtful. Even in cases where no restoration is to be hoped for, still we may mitigate the sufferings and prolong the life of the patient. Besides, although we may be satisfied that in the present state of our knowledge we cannot cure a given disease, we should not conclude that we shall never be able to cure it. Every physician should have for his motto, '*Nil desperandum.*' The general constitutional plan of treatment, before and after the development of tubercles, will not vary much. The same observance of hygienic rules will be necessary. Cold bathing will be less likely to be borne with benefit. More care in diet will be necessary. Medicine, though more necessary, will be less effectual.

The accumulation of tuberculous matter may be in any texture or in any organ of the body; but wherever situated, it constitutes scrofula, and is a fearful disease. Perhaps the most frequent seats are on the surface of the mucous membranes, in the parenchyma of the lungs, the lymphatic glands, the spleen, peritoneum and bones. Whilst we continue the treatment heretofore laid down, we must also be diligent in applications intended to benefit the local affection, especially if it should not appear that there are deposits in various parts. If the lymphatic glands should be most prominently affected, rubbing the surface over them with some of the ointments of iodine may be tried; or blisters over the gland, to be kept discharging and removed as often as may be necessary, will sometimes discuss the swelling. It will frequently be very difficult to say whether such swollen glands are truly tubercular, especially before fluctuation is evident. We must judge from the doughy feel of the tumor and the evidences of tubercular matter in other situations. When such deposit is found the means used to discuss the tumor will be less likely to succeed, because we have the presence of a foreign body to contend with. If such body is removed by the absorbents, it will require considerable time. If suppuration take place, after the matter is discharged we may still rub the adjacent parts with some of the preparations of iodine. We shall presently find a considerable portion of the surface of the tumor converted into an ulcer. This should be bathed with salt water, a solution of iodine, or one of creasote. If it should not improve under this treatment, it should have a blister laid on its surface for half an hour, or as long as the patient can conveniently bear it. This application should be repeated as may appear necessary. Dressing daily with an ointment of sulphate of copper, two scruples to the ounce, for a week, to be suspended for the same period and then repeated, will likewise be a proper dressing.

If the deposit should be in the lungs, in addition to the general treatment, we should institute counter irritation somewhere about the chest. This may be effected by blisters repeatedly applied, by pustulations with tartar emetic ointment, or by setons. Patients are very apt to get tired of blisters and pustulations. For that reason, perhaps, it would be better to rely on setons. Just here I may mention the use of *cod liver oil*, which has been highly extolled by Pereyra, Physician to Hospital St. Andrew, Bourdeaux, as a remedy in tubercular phthisis. It has also been considered very serviceable in other forms of scrofula. I do not doubt but it is a remedy suitable for scrofula, the more especially as it contains iodine in its composition. Whether all its virtues are dependent upon the iodine, or whether the iodine exists in a combination more adapted to benefit scrofulous disease, I am not prepared to say. We should observe, however, that the only case given by him, which I have seen, (Med. Chir. Rev., vol. 39, p. 503,) is one in which tubercular matter appears to have been confined to a small space in the lungs. He examined the patient three years after, when pectoriloquy still existed. Now allowing him full credit for the accuracy of his observations, (and to which indeed I think him fully entitled,) we should remember that such cases occasionally do well without any treatment. I know a young man who, some ten years ago, expectorated a considerable quantity of what, from description, I suppose to have been tubercular matter, in whom pectoriloquy of the right side is very evident, and who is in good health now. Whilst therefore we are disposed to allow due weight to each remedy, we should still be on the watch to see that we do not allow more than is due to some of them.

Wherever the deposits of tubercular matter may be, I hold it important that some mode of counter irritation be used at the same time, that we pursue the remedial and hygienic rules already noticed.

I will add a few words concerning that form of scrofula most common in this vicinity, I mean *Cachexia Africana.* During the prevalence of cholera, many negroes who appeared strongly predisposed to this disease were restored to a state of good health. This was effected, as I suppose, by the free use of calomel, which was then resorted to as a remedy for cholera. Now I believe that mercury must be used with caution in scrofulous diseases; and it certainly was not used with a sparing hand in those days. How is this to be accounted for? Thus: The beneficial action of a remedy does not depend upon the amount taken, but upon the effect produced. At that time our diseases required, at least tolerated, the use of calomel to an extent which would now do incalculable mischief. The quantity then used did not generally go beyond the remedial bounds; I say generally, for it is certainly true that even then great mischief was sometimes done. I think I have cured, at least that I have seen get well, divers cases of this disease, by pursuing the treatment advised, not by emetics, not by purgatives, not by tonics, but by what I thought a rational employment of each in its proper place, aided in no small degree by an enforcement of the hygienic regulations. I have always remembered that it was a work of time, and believed that the old adage 'the more haste, the less speed,' was peculiarly applicable.

A friend informed me that he had seen two cases cured, as he believed, by diuresis, in one case occasioned by taraxacum, in the other by a large quantity (two ounces) of vinegar of squills taken by mistake. Although diuresis frequently attends the employment of iodine, yet I am disposed to believe that the amendment in these cases was due to the previous judicious treatment which my friend had instituted; but the amendment becoming evident soon after an event so marked, there was an apparent fitness in ascribing it to that.

A long time ago, I was attending a case of this disease with very little satisfaction to myself, and no apparent advantage to my patient, when from some cause I ceased to attend. The mistress, thereupon, commenced giving a tincture of poke berries. (*Phytolacca Decandra.*) Soon after this course was adopted, the patient commenced improving. Whether such improvement was the delayed effect of my treatment, in whole or in part, I cannot tell. The remedy, however, is used in scrofulous affections by the same lady with, as she thinks, marked benefit.

A physician in one of our southern States, as I am informed, (but I have not learned his name or seen his book,) has written a book upon this disease, in which he considers walnut leaf tea *the remedy.* Negrier, of Angiers, some years ago, published a 'number of cases of disease of the lymphatics, with or without ulceration of the integuments, of scrofulous ophthalmia, of affections of the bones and periosteum, &c., in which a decided and very marked benefit was obtained from a course of this simply prepared tea. A handful of the fresh or slightly dried leaves may be added to a pint of boiling water, and of this infusion a small cupful may be taken twice a day. An extract may also be prepared by evaporation, and this Dr. Negrier recommends to be given at the same time either in the form of pills or of a thick syrup. A strong decoction of the leaves he has used with excellent effect as an application to scrofulous ulcers.' Med. Chirurg. Rev., vol. 36, p. 192—From Archives Generales. The reviewer 'feels inclined to predict that this remedy is one deserving notice, and that it will be found useful in some cases of lymphatic and cutaneous disease.' I am not informed as to the use or not of other means by Dr. Negrier. I cannot, therefore, express any opinion as to the proportion of credit due to the walnut leaves. I most sincerely wish that a remedy, an infallible remedy, may be found for this frightful disease; but I must confess that I expect the day to come at no great distance of time, when walnut leaf tea (at least when exclusively trusted to) will take its place by the side of *acorn coffee.*

Having spoken of poke berries and walnut leaves, I may perhaps as well name another remedy, which has been found successful in the cure of scrofula. I mean the prickly ash, (Xanthoxylum Fraxineum.) In West. Jour. Med. and Surg., vol. 4, p. 361, we have the report of three cases cured by this article. In each case a long course of treatment to no effect had preceded. It is directed to be prepared thus: boil a handful of the bark of the stem or root in a *new* iron vessel; let it stand until the iron has blackened the tea; give thrice daily as much as the stomach will bear without nausea. This shrub resembles little scrubby black locust when divested of leaves, and has a very pungent taste, which speedily imparts a tingling sensation through the nervous system, something like gum guiacum. I am very much disposed to believe that these cases were such as had been treated too long, and were in fact cases of mercurial disease instead of scrofula. Until better informed upon the subject, I shall place them on a footing with a very obstinate case of syphilis, which a medical friend of mine informed me had been effectually cured by May apple root (Podophyllum Peltatum). As a consequence, he had resolved to cure all cases of syphilis in the same way. It was suggested to him that, probably under the use of the May apple his friend had got well of the mercurial disease which had been substituted for the venereal. But he was so firmly persuaded of the virtues of the May-apple, that he put his next syphilitic patient upon it. But his good sense soon taught him the difference between May-apple and mercury.

*Conclusions.*—After such reflections as I have been able to make, I arrive at the following conclusions:

1st. That in a vast majority of instances scrofula owes its existence to inheritance; yet

2d. That there is no absolute necessity that a child having a scrofulous parent shall be scrofulous; on the contrary,

3d. That when one parent is scrofulous and the other not, a child which resembles the scrofulous parent will be much more apt to have scrofula than one which resembles the other parent; in fact, that the latter may have a well-grounded hope of escape.

4th. That the liability by inheritance depends upon a general, not upon a specific law, which is applicable to other diseases besides scrofula.

5th. That whilst a child born of scrofulous parents may escape, one born of parents not at all scrofulous, may have the disease.

6th. That scrofula depends upon an undue preponderance of the white parts of the blood, and the white tissues in the body.

7th. That in our treatment we should endeavor to restore a due proportion of the red particles to the blood, and of the red tissues to the body.

8th. That to effect this there is no specific; but we must be guided by general principles and rational views, precisely as is necessary to treat successfully any other disease.

9th. That how important soever medicine may be in the management of the disease, hygienic rules are by no means less so.

NOTE.—In all our quotations from the Medico-Chirurgical Review we have had reference to the American reprint of that work.

