# Errata

**Published:** 1846-12

**Authors:** 


### ERRATA.

Page 370, line 5 from bottom, for 'readers,' read 'reader.'
" 371 " 10 " " 'of train,' read 'train of.'
" 372 " 11 from top, for 'scurvy,' read scurfy.'
386, 425, and 427 pages, after 'Med. Chir. Rev., read 'new series.'
397 line 23, and 398 line 12, for 'purpura,' and 'purpuraceous,' read 'furfuraceous.'
Page 399, line 5 from top, for 'where,' read 'whose.'
" 400 " 8 " for 'suitable,' read 'situated.'
" 402 last line, for 'tathe,' read 'at the.'
" 405 line 33 from top, for 'enables,' read 'enable.'
" 406 " 27 " for 'appears,' read 'appear.'
" 415 " 3 " after esteemed, read 'as.'
" 415 last line, after diversity, read 'of opinion.'
" 416 line 21 from top, for 'varieties,' read 'variation.'
" 419 " 3 " for 'iodine,' read 'iodide.'
" 419 " 5 " for '$\frac{1}{52}$, $\frac{1}{54}$,' read '$\frac{1}{54}$, $\frac{1}{32}$.'
" 419 " 15 " for '$1\frac{1}{8}$,' read '$\frac{1}{18}$.'
" 424 " 23 " for 'removed,' read 'renewed.'

